# Suicide Prevention through Shared Information

**DOI:** 10.23889/ijpds.v1i1.359

**Published:** 2017-04-19

**Authors:** Lloyd Balbuena, Rudy Bowen, Marilyn Baetz

**Affiliations:** 1 University of Saskatchewan

## Objectives

Although mental health clinicians are in the best position to assess a person's risk for suicide, people at imminent risk may first seek the help of crisis workers, the police, hospital staff, or family members. The present project will use international (UK Biobank), provincial, and local crisis line data to elucidate imminent risks for suicide. An interdisciplinary team will discuss whether a common protocol for handling acute cases is warranted. The possibility of sharing a minimal set of information across services will also be discussed.

## Approach

The conceptual framework is that suicide is a probabilistic outcome of risks (comprised of inherited traits, habits, and environmental stressors) that can be put in temporal order as distal, proximate, and immediate antecedents. Local, provincial, and international data will be mined for risks corresponding to each epoch. The evidence will be assessed by an interdisciplinary team composed of patient advocates, psychiatrists, the police service, community health workers and academic researchers with the objective of reaching an agreement on a common protocol for suicidality assessment. The possibility of sharing a minimal dataset that is relevant to saving the lives of people at imminent risk of suicide will be explored. Finally, the efficacy of coordinated care across services will be evaluated by comparing suicide and self-harm rates will be assessed by comparing suicide and self-harm rates before and after the adoption of the protocol.

## Results

An interdisciplinary team has been formed and funding for the project is being sought. An application for data access to the UK Biobank received preliminary approval and is being evaluated by the scientific committee. Applications for access to provincial administrative data as well as telephone crisis line data for the last 10 years are being prepared.

## Conclusion

Routinely collected administrative data is a resource for the collective decision-making of an interdisciplinary team of experts and patient advocates. The ability of critical information to flow across organizational boundaries may be an important tool in suicide prevention. Dialogues regarding the ethical dilemma between potentially saving lives and potentially breaking privacy may need to happen.

